# Bisdemethoxycurcumin, a curcumin, protects chondrocytes, and reduces cartilage inflammation via the NRF2/HO‐1/NLRP3 pathway

**DOI:** 10.1002/iid3.1195

**Published:** 2024-02-27

**Authors:** Gang Jin, Wei Xu, Huilin Tang, Yaying Cui, Han Zhang

**Affiliations:** ^1^ Department of Orthopedics Taizhou Hospital Affiliated to Wenzhou Medical University, Linhai Zhejiang China

**Keywords:** bisdemethoxycurcumin, cartilage, Nrf2, osteoarthritis, pyroptosis

## Abstract

**Background:**

The objective of this thesis is to evaluate the effect of bisdemethoxycurcumin (BDMC) on osteoarthritis (OA) and comprehensively evaluate the role of the Nuclear Factor erythroid 2‐Related Factor 2 (Nrf2) signalling pathway in chondrocytes.

**Method:**

In our study, we treated chondrocytes with BDMC in an in vitro chondrocyte assay and measured its influence on extracellular matrix (ECM) expression, downstream heme oxygenase‐1 (HO‐1) and NOD‐like receptor thermal protein domain associated protein 3 (NLRP3) levels.

**Results:**

Our study indicates that BDMC significantly activates the Nrf2 signaling pathway in chondrocytes in vitro. Furthermore, the expression of matrix metalloproteinase 3, interleukin 1β, recombinant a disintegrin and metalloproteinase with thrombospondin motifs (ADAMTS)4 and (ADAMTS)5 was significantly suppressed by BDMC.

**Conclusion:**

This study confirms the potential for BDMC to activate the Nrf2/HO‐1/NLRP3 signalling pathway and alleviate OA symptoms. Therefore, BDMC is a promising therapeutic agent for OA that offers new insights and treatment methods.

## Graphical abstract

bisdemethoxycucurmin (BDMC) on osteoarthritis and to evaluate its regulatory role in the Nrf2 signaling pathway in chondrocytes, derived from the thyroid.

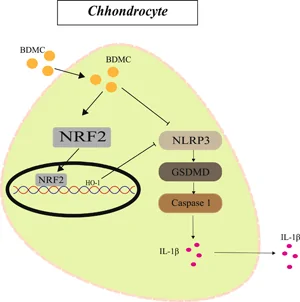

## 1 INTRODUCTION

Osteoarthritis (OA), a prevalent orthopedic ailment, is alternatively referred to as degenerative arthritis.^1^ Research findings indicate a substantial rise in the prevalence of knee OA,^2^, ^3^ accompanied by a corresponding escalation in the economic strain on individuals and the healthcare system.^4^ Joint pain and restricted range of motion are the main signs of OA.^5^ Age, gender, and obesity have been identified as major causative factors in addition to trauma.^4^ Yet, the precise cause of OA remains a mystery.^6^, ^7^ Most of the existing medications are symptom‐based and while they may provide temporary relief, they may also be associated with certain side effects.^8^ With this in mind, we can look for novel drugs that treat OA and have minimal side effects.

Oxidative stress is considered one of the primary causes of OA.^9^ The development of OA^10^, ^11^, ^12^, ^13^ may be triggered by activating matrix metalloproteinases (MMPs). This activation leads to the degradation of collagen II, aggrecan, and other extracellular matrix (ECM) components and ultimately destroys cartilage. The disintegrin and metalloproteinase with thrombospondin motifs (ADAMTS) plays a crucial role in this process. Tert‐Butyl hydroperoxide (TBHP) is a more stable form of hydrogen peroxide which can promote the production of Oxidative stress in vitro^14^ and has been widely used as an exogenous inducer of reticulum stress and reticulum stress‐related apoptosis of chondrocytes for in vitro studies on OA.^15^ The Nrf2/heme oxygenase‐1 (HO‐1) signaling pathway is vital in responding to oxidative stress and plays a key role in anti‐inflammatory, antioxidant, apoptotic, and other processes. Therefore, it is a major target for treating OA.^8^, ^13^ Inflammation not only causes oxidative stress but also accelerates it by activating proinflammatory pathways, including the well‐known NLRP3 inflammasome.^16^ The NLRP3 inflammasome is a molecular complex that oligomerizes and responds to various danger signals, such as ROS and interleukin 1β (IL‐1β).^17^, ^18^, ^19^ Its activation is accountable for regulating proinflammatory cytokines like IL‐1β and IL‐18, which bind to the adaptor protein, gasdermin D (GSDMD), activated by caspase‐1, and converted into mature forms.^20^, ^21^

The cytotoxic, antioxidant and anti‐inflammatory properties of Bisdemethoxycurcumin (BDMC) (also known as curcumin Ⅲ) have been demonstrated to be anti‐leukemia in colon, central nervous system, melanoma, kidney and breast cancer cell lines.^22^ BDMC treatment reduced serum NO and TNF‐α levels in angiogenesis‐induced animals. It has also been shown that BDMC can scavenge peroxynitrite to act as an antioxidant.^23^ Thus, studies described BDMC's antioxidant effects, and we investigated how BDMC modulates inflammation through NRF2/HO‐1/NLRP3. In this study, we investigated the potential anti‐inflammatory and anti‐ECM degradation effects of BDMC on mouse chondrocytes in vitro and investigated whether Nrf2/HO‐1/NLRP3 cascades are involved in such effects.

## 2 MATERIALS AND METHODS

### 2.1 Reagents and antibodies

MCE provided BDMC (#HY‐N0007, purity ≥ 98%). GLPBIO provided TBHP (#GB30288, purity ≥ 98%). Primary antibodies against ADAMTS4 (#ab179475, 1:1000), ADAMTS5 (#ab179475, 1:100), MMP3 (#ab76110, 1:100), MMP9 (#ab124995, 1:100), and HO‐1 (#ab68477, 1:1000) were obtained from Abcam, while Cell Signaling Technology supplied β‐Actin (#3700, 1:1000). Nrf2 (#ER1706‐41, 1:1000) and secondary antibody Alexa Fluor 594 (#HA1121, 1:200) were products of Huabio. The primary antibody specific for collagen II (#K009364P, 1:500) was obtained from Solarbio, while the primary antibody against aggrecan (#PA1‐1746, 1:1000) and the primary antibody against IL‐1β (#A1112, 1:1000) were obtained from Thermo Fisher Scientific and ABclonal Technology, respectively. The secondary antibody (#S0001, 1:20000) was supplied by Affbiotech.

### 2.2 Molecular docking

Molecular docking, a computational technique, verifies the binding activity of compounds to essential targets. The present study utilized AutoDock Vina (Vina, 1.1.2), a semi‐flexible program accurately measuring a rate of 78%. The target crystal structure for NLRP3 with PDBID 2naq was secured from the Protein Data Bank (PDB) RCSB, and the structure of BMDC was obtained from the PubChem database. PyMOL version 4.3.0 software was employed for the separation of the original structures of the ligand and protein, dehydration, and elimination of any organic substance. AutodockTools was used thereafter for hydrogenation, assessment of charge, atomic type designation (as AD4 type), gustator calculation, and creation of the docking grid box for the protein structure. Determination of the chemical composition (small molecule ligand) of the root is vital to selecting the twistable bond of the ligand. To enable future docking, it is imperative to convert the protein structure and small molecule ligand formats from “PDB” to “PDBQT” utilizing AutodockTools. The outcomes were assessed and visualized using the PLIP online website and Discovery Studio analysis software in terms of 3D forces and 2D angles.

### 2.3 Cultivation of ATDC5 cells

The ATDC5 chondrocyte cell line demonstrates the chondrocyte phenotype. The cells were cultured in DMEM with a 94% concentration, supplemented by 5% fetal bovine serum and 1% penicillin‐streptomycin. Afterward, all cell cultures were incubated for cell passaging once or twice within 2 days at 37°C and 5% CO_2_ in a humidified incubator. The cells were then harvested for experiments once they reached 80%−90% confluence.

### 2.4 Cell viability assay

The CCK‐8 assay was used to evaluate the impact of BMDC on the activity of ATDC5 cells. 96‐well plates (5000 cells/well) were seeded with ATDC5 cells and treated with BMDC at various concentrations (0, 1, 2, 4, 8, 16, 32, 64, 128, and 256 μM) for either 24 or 48 h. Next, 10 µL of CCK‐8 was added to each well, followed by incubation at 37 degrees Celsius for an additional 2 h. The OD density was then measured using a Thermo Fisher spectrophotometer at 450 nm according to the manufacturer's instructions. All assays were performed in triplicate. CCK‐8 reagent (10 µL) was added to 96‐well cell culture plates that contained the cultures and were then incubated for 1.5 h in 5% carbon dioxide at 37°C. The optical density of each well was measured utilizing a Multiskan FC Microplate Photometer from Thermo Fisher Scientific at a wavelength of 450 nm.

### 2.5 Quantitative RT‐PCR experiments

Total RNA was extracted from chondrocytes using the RNA Extraction Kit (Beyotime) and stimulated with different concentrations of BMDC and TBHP (20 ng/mL). Reverse transcriptase was used to synthesize cDNA from 500 ng of total RNA. For quantitative RT‐PCR (qPCR), a SYBR master mix of x2 consisted of 5 μL, 0.2 μL primer, 2 μL ddH2O, and 2 μL diluted cDNA, and was used, resulting in a total reaction volume of 10 μL. This experiment was conducted using an ABI 7500 Real‐Time PCR Detection System (Applied Biosystems). Table 1 includes a list of the primers used for the detection of the target gene.

**Table 1:** The primers.

Gene	Primer sequences (5−3)	
Collagen2	Forward	TGACCTGACGCCCATTCATC
	Reverse	TTTCCTGTCTCTGCCTTGACCC
Aggrecan	Forward	TCACCATCCCCTGCTACTTCATC
	Reverse	TCTCCTTGGAAATGCGGCTC
ADAMTS‐4	Forward	AACTCGAAGCAATGCACTGGT
	Reverse	TGCCCGAAGCCATTGTCTA
ADAMTS‐5	Forward	GCATTGACGCATCCAAACCC
	Reverse	CGTGGTAGGTCCAGCAAACAGTTAC
MMP9	Forward	CGACTTTTGTGGTCTTCCCC
	Reverse	TGAAGGTTTGGAATCGACCC
MMP3	Forward	CAGGTTCGCCAAAATGGAGG
	Reverse	CAGCCTTGGCTGAGTGGTAGA
GAPDH	Forward	ACCCAGAAGACTGTGGATGG
	Reverse	CACATTGGGGGTAGGAACAC

### 2.6 Western blot analysis

The RIPA lysis buffer (Cell Signaling Technology) utilized for lysing ATDC5 cells had phenylmethylsulphonyl fluoride protease inhibitor added to it using the BCA kit obtained from AMEKO in Shanghai, China. Equal amounts (20 µg) of protein were subjected to 10% sodium dodecyl sulfate‐polyacrylamide gel electrophoresis for separation. The proteins were transferred onto a polyvinylidene difluoride (PVDF) membrane (Bio‐Rad) using electroblotting. The membrane was blocked for 1 h at room temperature (RT) with a 5% solution of bovine serum albumin (BSA) (Biosharp). Then, it was incubated with a specific primary antibody (dilution of 1:1000) for 8−12 h at 4°C. Technical abbreviation explanations will be provided upon their first use. The PVDF membrane received three washes, each 5 min long, with TBST. It was then incubated with a compatible HRP‐conjugated secondary antibody for 1 h at RT on a shaker. After three additional 5‐min washes with TBST, the protein‐antibody complexes were detected using Enhanced Chemiluminescence Reagent (Millipore). The ImageQuant LAS‐500 Imaging System from GE Life Sciences in Chicago, IL was utilized for imaging purposes, while protein band densities were assessed with the aid of ImageJ software from the NIH in Bethesda, MD.

### 2.7 Immunofluorescence staining

Slides in a 6‐well plate were used to culture ATDC5 cells. Upon reaching 20% confluence, IL‐1β and 4 nM were either used to stimulate or to deactivate the cells for 24 h at 37°C. The cells were fixed with 4% PFA for 20 min and washed thrice with PBS (pH 7.4) at RT. Subsequently, the cells were exposed to a 0.2% Triton X‐100 solution for 15 min. After incubating with 5% BSA for 1 h at 37°C, the cells were subsequently incubated with antibodies overnight at 4°C. The primary Anti‐AD5 antibody was diluted to a ratio of 1:100. Following this, the cells were exposed to either Alexa Fluor 488 or Alexa Fluor 594 fluorescent antibodies for 1 h in the dark at RT. After that, 4',6‐diamidino‐2‐phenylindole (DAPI) (Biotime, Shanghai, China) was added and incubated for 5 min in the dark at RT. Random samples were taken using fluorescence microscopy (Olympus).

### 2.8 High‐density cell culture

At 37°C and under 5% CO_2_, culture conditions were maintained using 94% DMEM, 5% fetal bovine serum, and 1% penicillin‐streptomycin. The medium was changed every other day. In all experiments, the concentration of dimethyl sulfoxide was kept below 1:1000. To attain a high‐density culture, ATDC5 cells were seeded at a density of cells/mL by inoculating 10 µL in a 24‐well plate and were allowed to adhere for 6 h. Following this, the necessary drugs and culture medium were introduced, and the cells were incubated for 7 days, with the medium being replenished every 2 days. The cells were then fixed using paraformaldehyde and stained according to instructions provided by the toluidine blue staining kit. Subsequently, we scanned and photographed the 24‐well plate using an EPSON V600 photo scanner from Japan.

### 2.9 Histology and immunohistochemistry

After undergoing decalcification in a 10% EDTA solution, the calcified bone tissue was fixed and subsequently immersed in a series of ethanol solutions with concentrations of 75%, 85%, 95%, and 100%. The tissue was cleared in xylene and embedded in paraffin blocks. The sections were then stained with hematoxylin and eosin (H&E) and Safranin O‐fast green (SO) for visualization. H&E and SO staining solutions were applied to dewaxed tissue sections for 3 min. The tissue sections were ultimately dehydrated in isopropyl alcohol and mounted.

### 2.10 Statistical analysis

GraphPad Prism software (GraphPad Software Inc.) was used to analyses group differences. The figures depict the mean ± standard deviation of at least three separate experiments. Significant differences were indicated with *p* < .05.

## 3 RESULTS

### 3.1 Identification of affinity between BMDC and NLRP3

**Figure 1:**
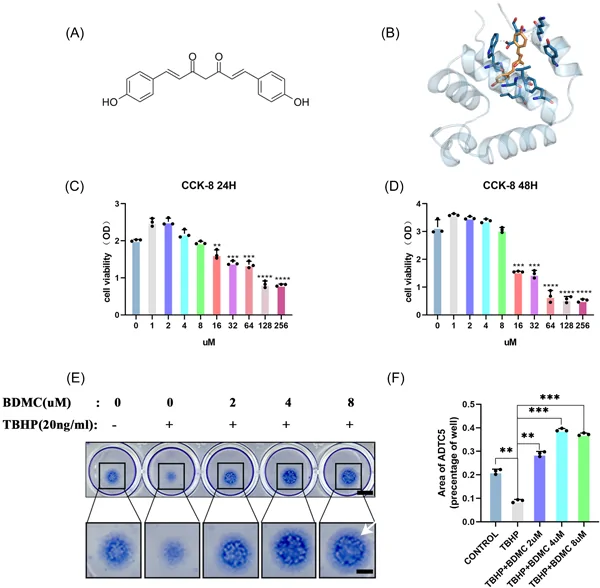
Low concentrations of BDMC were not cytotoxic to ATDC5 cells and inhibited TBHP induced cell diminution in vitro. (A) Chemical structure of PD032590. (B) The 2D structure image of BDMC interacting with NLRP3 in the image. (C) Cell counting Kit 8 assay results of BMMs stimulated with BDMC at different concentrations (0, 1, 2, 4, 8, 16, 32, 64, 128, and 256) nM and over different time periods (24 and 48 h). (E) Toluidine blue staining of ATdc5 chondrocytes using high‐density culture, stimulated with TBHP (10 ng/mL) or/and BDMC (2, 4, and 8 nM) for 7 days. Scale bar:5 mm/2.5 mm. (F) Ratio of area of ATDC5 and total integrated density was analyzed using Image J. All data are presented as the mean ± Sd from three experiments. *p < .05, **p < .01, ***p < .001, ****p < .0001.

The molecular formula of BMDC is shown in Figure 1A. For subsequent experiments, we developed a model to analyze the interaction between BMDC and NLRP3 (as illustrated in Figure 1B). Our results demonstrate that BMDC interacts with the receptor protein NLRP3 through two hydrophobic forces, measuring 3.1 and 3.7 Å respectively, at amino acid residue 73 PHE. Additionally, two hydrophobic forces, measuring 3.3 and 3.4 Å respectively, bind to amino acid residue 8 of LEU at the receptor protein NLRP3. The two hydrophobic forces of 3.1 and 3.7 Å also exhibit a binding affinity to amino acid residue 70 VAL of the receptor protein NLRP3. Binding to amino acid residue 12 of LEU and amino acid residue 86 of LYS of the receptor protein NLRP3 occurs through 2 hydrophobic forces, measuring 3.4 and 3.7 Å, respectively. Two hydrophobic forces of 3.9 Å each enabled binding of NLRP3's receptor protein to amino acid residues 82 TYR and 66 TRP. In addition, two‐dimensional interaction force analysis yielded similar results. The stabilization of the binding between NLRP3, the target protein, and BMDC, the compound, is a result of these forces.

### 3.2 BMDC promotes cartilage differentiation in vitro

The cytotoxicity of BMDCs was evaluated on ATDC5 cells, which are a well‐known in vitro model used in the study of endochondral ossification, by utilizing the CCK‐8 assay. BMDC's CC50 value is 14.62 nM after 48 h, and BMDC above 8 nM has obvious toxic effects on ATDC5. To prevent any further harm to the cells, a drug concentration below 8 nM was utilized (Figure 1C,D). Then we confirmed through high‐density culture that treatment of ATDC5 cells with BMDC stimulated their proliferation and differentiation after being stimulated with TBHP at either 0 or 20 ng/mL (Figure 1E,F).

### 3.3 BDMC protects the ECM of TBHP‐stimulated chondrocytes

**Figure 2:**
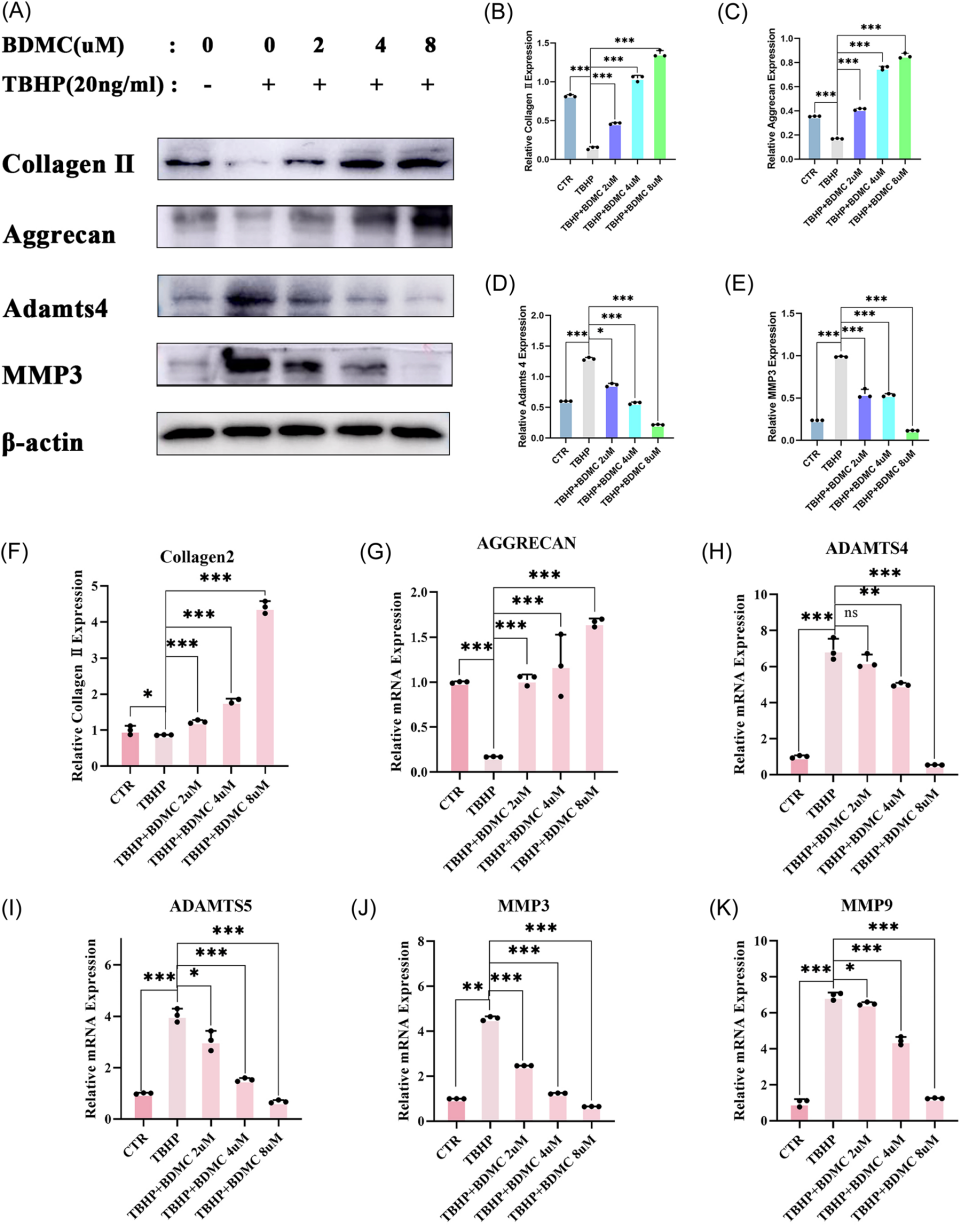
BDMC inhibits ECM proteolysis in TBHP‐stimulated ATDC5 cells. (A−E) Immunoblotting was performed to analyze Collagen II, Aggrecan, Adamts4, MMP3 and GAPDH expression. Protein expression was assessed using Image J. (F−K) qRT‐PCR was used to assess the effect of TBHP on Collagen II, Aggrecan, Adamts4, Adamts5, MMP3, and MMP9 mRNA expression. All data are expressed as the mean of three experiments ± Sd. *p < .05, **p < .01, ***p < .001, ****p < .0001.

Based on the results, cells were either left untreated (ATDC5) or stimulated with 20 ng/ml TBHP, 0.2, 4, or 8 nM BDMC for 24 h. The expression of cartilage ECM was detected using a protein blotting assay. The phenotypic marker proteins, COL2 and aggrecan, were upregulated and MMP9, MMP3, ADAMTS5, and ADAMTS4 were inhibited in ATDC5 cells (Figure 2A−E), after 48 h. Matrix metalloproteinases, specifically MMP3 and MMP9, degrade Type II collagen, while ADAMTS4 and ADAMTS5 metalloenzymes degrade aggrecan. To investigate how BDMC regulates ATDC5 cells, the expression of phenotypic marker genes collagen II, aggrecan, matrix metalloproteinase 3, MMP‐9, ADAMTS4, and ADAMTS5 were analyzed. At the gene level, BDMC showed protective effects against collagen 2 and aggrecan and inhibitory effects against MMP9, MMP3, ADAMTS4 and ADAMTS5. To sum up, BDMC stimulated the proliferation and differentiation of ATDC5 cells in a laboratory setting (Figure 2F−K).

### 3.4 BDMC activates the Nrf2/ho‐1 signaling pathway in ATDC5 cells stimulated with TBHP, resulting in inhibition of cellular pyroptosis

**Figure 3:**
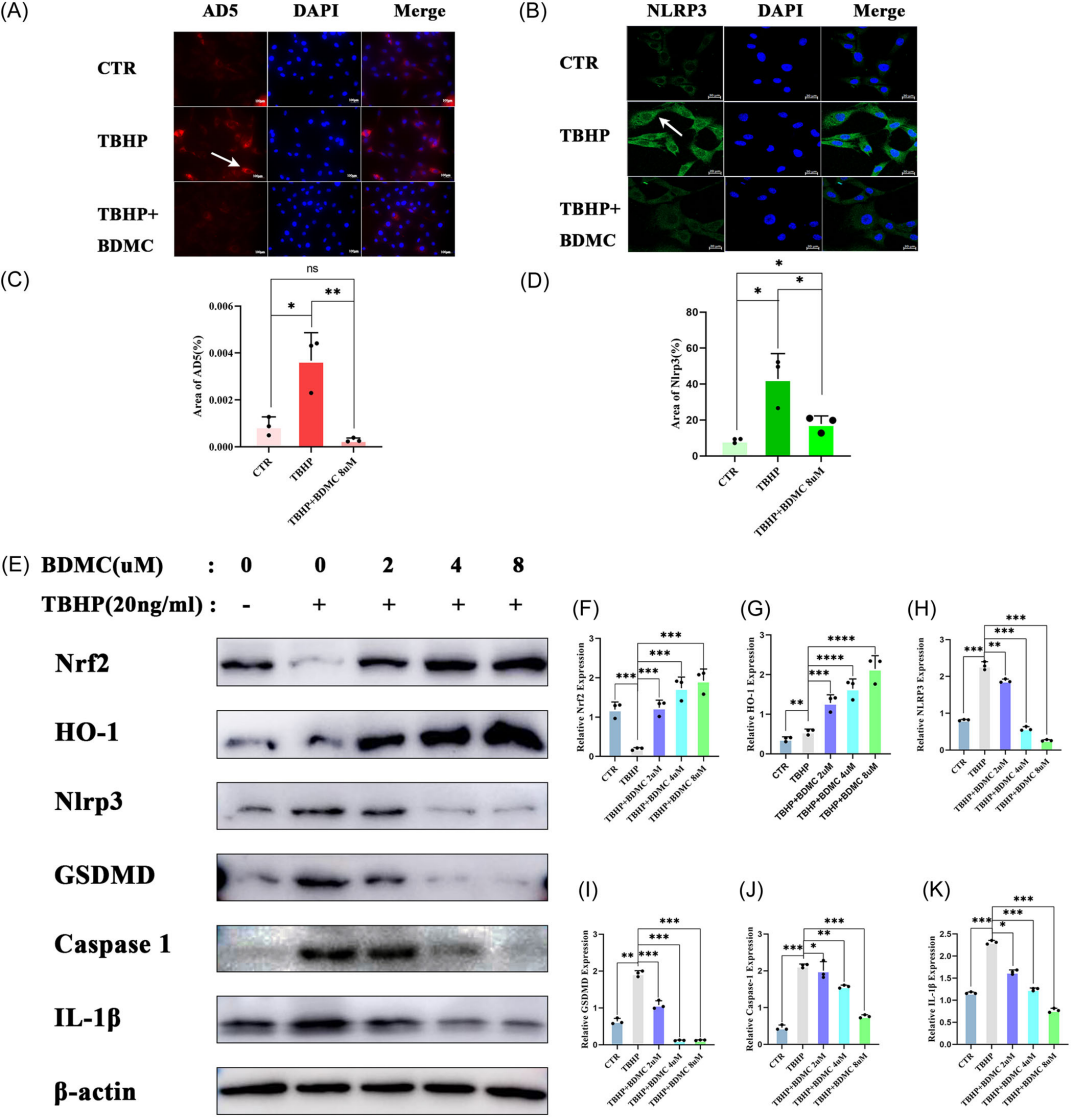
PD0325901 activated the NRF2/HO‐1 pathway and pyroptosis in TBHP—stimulated ATDC5 cells. (A−D) Protein expression of ADAMTS5 and NLRP3 was assessed by cellular immunofluorescence (scale bar: 100 μm/20 μm) and using Image J. The protein expression of ADAMTS5 and NLRP3 was evaluated in TBHP ‐stimulated ATDC5 cells. (E−K) Western blot analysis of Nrf2, HO‐1, NLRP3, GSDMD, Caspase 1, L‐1β and GAPDH expression in TBHP (20 ng/mL) stimulated ATDC5 cells. All data are expressed as the mean ± Sd of three experiments.*p < .05, **p < .01, ***p < .001, ***p < .0001.

To further explore the mechanism by which BDMC regulates anti‐inflammatory and protective ECM in ATDC5 cells, we found by immunofluorescence that BDMC inhibited the upregulation of Adamts5 (Figure 3A,B). We then further examined the immunofluorescence of NLRP3 and found that BDMC simultaneously reduced the production of pyroptotic vesicles in ATDC5 cells (Figure 3C, D). Western blot analysis of Nrf2/HO‐1 and various proteins involved in cell death in ATDC‐5 cells (Figure 3E−K) revealed that BDMC rescued Nrf2/HO‐1 inhibited by TBHP stimulation and partially inhibited the production of Nlrp3, GSDMD, caspase 1, and IL‐1β. These experimental findings demonstrate that BDMC can inhibit cellular pyroptosis induced by Nlrp3 activation through activating the Nrf2/HO‐1 pathway within the cell.

## 4 DISCUSSION

**Figure 4:**
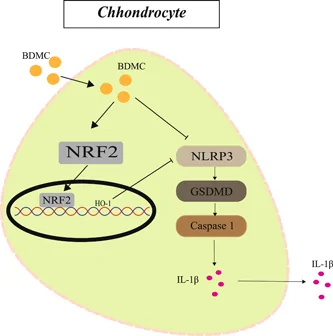
Schematic representation of OA inhibition by BDMC. BDMC inhibits the pyroptosis pathway by activating the Nrf2/HO‐1 signaling pathway, which exerts an effect on the degradation of cartilage ECM. BDMC, Bisdemethoxycurcumin; ECM, extracellular matrix; OA, osteoarthritis.

Studies have demonstrated that inflammation and ECM deterioration are essential for the emergence of OA.^24^, ^25^ Nonsteroidal anti‐inflammatory drugs (NSAIDs) are currently the most commonly prescribed medications for the treatment of OA and are used primarily for the relief of symptoms. However, these medications do not prevent the progression of OA, and can have a range of adverse effects, such as heightened cardiovascular strain and gastrointestinal reactions.^26^, ^27^, ^28^ Therefore, the identification of drug candidates for OA treatment is essential. Our study discovered that BDMC effectively inhibited inflammation and ECM degradation in chondrocytes induced by TBHP. The Nrf2/HO‐1/NLRP3 pathway was demonstrated to be a factor in the amelioration of OA pathogenesis in vitro by BDMC (Figure 4).

It has been reported that Nrf2 deficiency increases susceptibility to inflammatory diseases, whereas Nrf2 activation promotes expression of the downstream gene HO‐1, inhibits NLRP3 activation in pyroptosis vesicles, and reduces inflammation and ECM degradation.^29^, ^30^, ^31^, ^32^ Therefore, we hypothesized that Nrf2 activated by BDMC could have a compensatory role in OA. This study demonstrated the critical role of Nrf2/HO‐1 in anti‐inflammatory and ECM degradation, as depicted in Figure 3E. In conclusion, Nrf2/HO‐1 is a crucial factor in anti‐inflammation and ECM degradation. BDMC augmented Nrf2 expression, upregulated HO‐1 expression, and downregulated focal inflammatory factors NLRP3 and IL‐1β. In conclusion, Nrf2/HO‐1 is a crucial factor in anti‐inflammation and ECM degradation.

We investigated whether BDMC can alleviate OA through alternative pathways. Myd88, a major effector molecule in Toll‐like receptors (TLRs) signaling,^33^ has been observed to be activated by IL‐1β in prior studies.^34^ Upon recognition of a ligand, TLRs trigger downstream signaling through Myd88, subsequently activating nuclear factor‐κB (NF‐κB) and increasing expression of inflammatory cytokines. Nrf2 has also demonstrated its role in regulating the TLR‐4/MyD88/NF‐κB signaling pathway during certain pathological processes.^35^, ^36^ Therefore, BDMC may exert its effects through the Nrf2/MyD88/NF‐κB pathway. Recent research has demonstrated that Nrf2 activation may impede inflammation‐induced harm through its antioxidant pathway, necessitating additional investigations to validate.^37^

Our study revealed that BDMC had an effect on the inflammatory response of chondrocytes and promoted ECM homeostasis. However, there are several limitations to our research. To demonstrate its in vivo efficacy against OA, further characterization and functional studies in animal models such as the destabilization of the medial meniscus (DMM) model must be conducted.^38^ In particular, an assessment of BDMC's impact on Nrf2 knockout mice is warranted.^39^, ^40^ Previous studies have reported that BDMC can be successfully encapsulated in liposomes, which could greatly enhance the absorption of BDMC at the joint.^41^ Recently, a novel joint health formula containing BDMC enriched curcumin, 3‐O‐Acetyl‐11‐keto‐beta‐Boswellic acid‐enriched curcumin, and Ashwagandha demonstrated a protective effect against OA. Boswellia, and Ashwagandha demonstrated a protective effect against OA,^42^ which suggests that instead of single use of BDMC in subsequent in vivo experiments in animals, we can adopt the novelty of the experiment in the form of multiple herbal complexes encapsulated in liposomes The novelty of the experiment will be greatly improved. And these findings do not indicate that incorporating ginger into one's diet can replace the use of smaller doses of purified curcumin. Therefore, further research is needed to establish this relationship.

In conclusion, our study revealed that BDMC effectively inhibits NLRP3 through the activation of the Nrf2/HO‐1 pathway in chondrocytes, resulting in a significant reduction of IL‐1β‐induced inflammatory responses and catabolism. Our in vitro models further demonstrated that BDMC significantly enhances Nrf2 and HO‐1 expression, thus reversing ECM degradation and inflammatory responses. Our research suggests that BDMC could be a promising treatment for OA due to its antioxidant effect primarily resulting from Nrf2 activation. However, the inhibitory effect it had on TBHP‐induced inflammation could be dependent on Nrf2 activation or independent of it.

## AUTHOR CONTRIBUTIONS

The manuscript was composed of Gang Jin, Wei Xu., and Yaying Cui, while Wei Xu and Yaying Cui conducted the experiments, Huilin Tang generated the figures, Yaying Cui revised and down‐weighted the article, and Han Zhang was integral to the entire process, including the submission of the manuscript.

## CONFLICT OF INTEREST STATEMENT

The authors declare no conflict of interest.

## Data Availability

Data openly available in a public repository.
